# Pharmacodynamic/Pharmacogenomic Modeling of Insulin Resistance Genes in Rat Muscle After Methylprednisolone Treatment: Exploring Regulatory Signaling Cascades

**DOI:** 10.4137/grsb.s613

**Published:** 2008-04-23

**Authors:** Zhenling Yao, Eric P. Hoffman, Svetlana Ghimbovschi, Debra C. DuBois, Richard R. Almon, William J. Jusko

**Affiliations:** 1 Department of Pharmaceutical Sciences, School of Pharmacy and Pharmaceutical Sciences; 2 Department of Biological Sciences, State University of New York at Buffalo, Buffalo, New York; 3 Children’s National Research Center, Washington D.C

**Keywords:** corticosteroid, glucocorticoid, microarrays, mathematical modeling, insulin resistance

## Abstract

Corticosteroids (CS) effects on insulin resistance related genes in rat skeletal muscle were studied. In our acute study, adrenalectomized (ADX) rats were given single doses of 50 mg/kg methylprednisolone (MPL) intravenously. In our chronic study, ADX rats were implanted with Alzet mini-pumps giving zero-order release rates of 0.3 mg/kg/h MPL and sacrificed at various times up to 7 days. Total RNA was extracted from gastrocnemius muscles and hybridized to Affymetrix GeneChips. Data mining and literature searches identified 6 insulin resistance related genes which exhibited complex regulatory pathways. Insulin receptor substrate-1 (IRS-1), uncoupling protein 3 (UCP3), pyruvate dehydrogenase kinase isoenzyme 4 (PDK4), fatty acid translocase (FAT) and glycerol-3-phosphate acyltransferase (GPAT) dynamic profiles were modeled with mutual effects by calculated nuclear drug-receptor complex (DR(N)) and transcription factors. The oscillatory feature of endothelin-1 (ET-1) expression was depicted by a negative feedback loop. These integrated models provide testable quantitative hypotheses for these regulatory cascades.

## Introduction

A major function of the hypothalmic/pituitary/adrenal axis (HPA-axis) involves regulating the production, storage, use, and distribution of substrates for systemic energy metabolism. Glucocorticoids, the effecter hormones from the adrenal gland, were named for their glucose-regulating properties and have extensive impact on carbohydrate, lipid, and protein metabolism. Glucocorticoids are the major regulators of systemic gluconeogenesis, a multi-tissue phenomena involving substrate flow from muscle and fat coupled with enzymatic synthesis of glucose from these substrates in liver and kidney. Glucocorticoid output from the adrenal cortex, which occurs with a circadian periodicity, serves to maintain adequate blood glucose in order to assure that sufficient glucose is available to fulfill the needs of vital organs, particularly the brain. This is accomplished by adjusting both supply and demand. On the supply side, glucocorticoids increase the synthesis and activity of enzymes in the gluconeogenic pathway in liver and kidney, as well as ancillary enzymes/pathways such as expression of aminotransferases and components of the urea cycle. They also control the release of gluconeogenic substrates from muscle and fat. On the demand side, they decrease glucose utilization in peripheral tissues which in part encompasses their anti-inflammatory and immunosuppressive actions.

Synthetic glucocorticoids, corticosteroids (CS), are used therapeutically for a wide variety of conditions that require immune and/or inflammatory modulation. Because corticosteroids pharmacologically magnify the physiological actions of glucocorticoids, therapeutic use of this class of drugs is accompanied by a wide range of adverse effects that include muscle wasting and hyperglycemia. Skeletal muscle is the major organ responsible for glucose disposal, accounting for about 80% of total glucose uptake under insulin stimulation. Corticosteroids exert their glucose sparing effect on skeletal muscle mainly by inhibiting glucose utilization and switching muscle energy metabolism from glucose to lipids. Inhibition of insulin-directed glucose disposal in skeletal muscle in conjunction with enhanced gluconeogenesis results in elevated plasma glucose levels (diabetes). In addition, prolonged exposure to corticosteroids and the attendant net degradation of muscle protein causes atrophy of the musculature.

Previously, we used a rat model and employed Affymetrix gene arrays to profile the time course of changes in gene expression in skeletal muscle of male rats in response to a single bolus dose of the synthetic corticosteroid, methylprednisolone (MPL). ADX animals were used to eliminate circadian oscillation in corticosteroid responsive genes, which facilitated data mining. This time course involved 16 time points over a 72 hour period plus untreated controls. Because these experiments were initiated using adrenalectomized animals, the drug in effect acted as a stimulus that perturbed the homeostatic balance of the system, and the experiment monitored the deviation of the system and its return to the original state. The objective was to identify those changes in transcription induced by corticosteroids, which if perpetuated by repeated dosing, would manifest as chronic adverse effects. Using a filtering approach to eliminate unregulated probe sets, we identified 653 probe sets out of 8799 with a high probability of being regulated (Almon et al. 2004). Of these, we identified 29 probe sets (representing 22 gene transcripts) directly related to insulin resistance (Almon et al. 2005b).

Although very useful, a single time series only provides a one dimensional view of the dynamics of the system in response to the stimulus. A pharmacological time series is different from most time series studies (for example those assessing developmental changes) in that it can be challenged using a different dosing regimen. A second dosing regimen is valuable in two regards. First, it can serve to corroborate results of the first dosing regimen. If changes in expression levels for a particular transcript are observed in multiple time points and in multiple animals from two different doses, these changes are unlikely to result from chip artifacts. Second, the results can be used to group genes with common mechanisms of regulation. If two or more genes have a common mechanism of regulation, then their response profiles should follow the same pattern regardless of the dosing regimen. More recently, we used microarrays to broadly characterize the response of skeletal muscle to a second dosing regimen which entailed chronic infusion of MPL in a group of ADX male rats. For these experiments MPL was infused at 0.3 mg/kg/h for 168 hours using Alzet osmotic pumps. Four animals were sacrificed at each of ten different times during the infusion period. Here the drug essentially was used as an unbalancing stimulus and the experiment evaluated the capacity of the system to rebalance in the continuous presence of the drug.

Microarrays provide a method of high throughput data collection that is necessary for constructing comprehensive information on the transcriptional basis of such complex systemic polygenic phenomena as insulin resistance. When microarrays are used in rich in vivo time series studies they yield temporal patterns of changes in gene expression that illustrate the cascade of molecular events in time that cause complex responses. With such data, the challenge then becomes one of constructing rational, quantitative, mechanism-based frameworks that describe the relationships between the elements of the cascades. Kinetic/dynamic modeling provides an approach to developing quantitative, testable, mechanism-based hypotheses concerning the relationship between drug kinetics and elements of cascades that continue long after the drug has dissipated. Such models can accommodate hierarchal cascades where one process generates molecules that become effectors or mediators for other processes. They can also accommodate convergence of cascades that commonly occur in the control of the expression of genes where binding sites for multiple nuclear factors participate in the regulation of the level of expression of a particular mRNA. Proteins (transcription factors) expressed from this mRNA in turn become endogenous mediators that may themselves become effector molecules for other processes.

In the present report we use the two response profiles (acute and chronic) to develop kinetic/dynamic models for the regulation of the expression of six genes that contribute to corticosteroid induced insulin resistance in skeletal muscle. The first is insulin receptor substrate-1 (IRS-1). Here we model the expression profile as being driven by both MPL and Interleukin 6 (IL-6). The second and third are two nuclear encoded mitochondrial genes with similar expression profiles, uncoupling protein 3 (UPC3) and pyruvate dehydrogenase kinase isoenzyme 4 (PDK4). These two genes are modeled as being driven by both MPL and the muscle specific transcription factor MyoD. The fourth and fifth are two genes related to the increased use of lipids for energy, fatty acid translocase (FAT) and glycerol-3-phosphate acyltransferase (GPAT). These two genes are modeled as being under the combined influence of both MPL and sterol regulatory element binding protein-1c (SREBP-1c). The last is endothelin-1 (ET-1) which influences vascular resistance in skeletal muscle, thereby influencing both blood flow and glucose disposal. Although these are four separate models, they represent a beginning at developing more comprehensive models for the complex phenomena of glucocorticoid-induced insulin resistance in skeletal muscle. Such models gain more importance when the data linking glucocorticoids to the insulin resistance associated with metabolic syndrome are considered.

## Materials and Methods

### Experimental

Gastrocnemius muscles were obtained from animal studies performed in our laboratory (Sun et al. 1999; Ramakrishnan et al. 2002). In the acute study, male ADX Wistar rats weighing 225–250 g (Harlan Spague-Dawley Inc., IN) were subject to right external jugular vein cannulation under light ether anesthesia one day prior to the study. A single intravenous bolus of 50 mg/kg MPL (Solu-Medrol, Pharmacia-Upjohn Company, MI) was given to 47 animals via right jugular vein cannula. Rats were sacrificed by aortic exsanguination at 0.25, 0.5, 0.75, 1, 2, 4, 5, 5.5, 6, 7, 8, 12, 18, 30, 48 and 72 h after dosing. The sampling time points were selected based on previous studies of receptor dynamics and enzyme induction in muscle and liver (Sun et al. 1998; Sun et al. 1999; Ramakrishnan et al. 2002). Four vehicle-treated rats were designated as controls and were considered as sacrificed at time point zero. In a companion study, 6 animals were treated with single intravenous bolus of 50 mg/kg MPL. Blood samples were taken serially from the cannula at 0.5, 1, 2, 4, 5, 6, 8, 10, 12, 24, 36, 48 and 72 h. Blood samples were stored at −80 °C and were used to determine plasma MPL concentrations. In our chronic study, 40 male ADX Wistar rats weighing 300–350 g were given 0.3 mg/kg/h infusions of MPL reconstituted in supplied diluent. The infusions were given using Alzet osmotic pumps (Model, 2001, flow rate 1 ul/h; Alza, Palo Alto, CA). The pump drug solutions were prepared for each rat based on its pre-dose body weight. Pumps were subcutaneously implanted between the shoulder blades on the back. Four rats were sacrificed by aortic exsanguination at 6, 10, 13, 18, 24, 36, 48, 72, 96, and 168 h. Four control rats were implanted with a saline-filled pump and sacrificed at various times throughout the 7-day study period. Gastrocnemius muscles were rapidly excised, quickly frozen in liquid nitrogen, and stored at −80 °C.

### Drug assay

MPL plasma concentrations were measured by a previously described normal-phase high performance liquid chromatography (HPLC) method (Haughey and Jusko, 1988). The quantitation limit of this method is 10 ng/ml.

### RNA extraction

Frozen gastrocnemius muscles were ground into powder using a mortar and pestle chilled with liquid nitrogen. Total RNA extractions were carried out by a Trizol-chloroform based extraction method. About 100 mg muscle powder from each animal was added to prechilled Trizol Reagent (Invitrogen, Carlsbad, CA) at a ratio of tissue: Trizol of 1:10. Extractions were performed according to manufacturer protocols. Extracted RNA was further purified by passage through RNUeasy mini-columns (Qiagen, Valencia, CA). Final extracted RNA samples were resuspended in nuclease-free water. Quantity of total RNA was determined by spectrophotometry and purity was assessed by agarose gel electrophoresis. Extracted total RNA preparations were stored at −80 °C.

### Microarrays

Isolated muscle RNA from each individual animal was used to prepare biotinylated cRNA target according to manufacturer protocols. Then biotinylated cRNAs from the acute study were hybridized to 51 individual Affymetrix GeneChip^®^ Rat Genome U34A (Affymetrix, Santa Clara, CA), which contained 8799 probe sets. The target cRNAs from the chronic study were hybridized to 44 individual Affymetrix GeneChip^®^ Rat Genome 230A (Affymetrix, Santa Clara, CA), which contained 15967 probe sets. Oligonucleotide microarrays were utilized because of their high reproducibility between separate arrays. Both datasets have been submitted to the National Center for Biotechnology Information (NCBI) Gene Expression Omnibus database (GSE490 and GSE5101) and are also available online at http://pepr.cnmcresearch.org/ (Almon et al. 2003). Our approach to identifying probes of interest has been described in previously published articles on mining the datasets for muscle, liver and kidney from the acute MPL treated animals (Almon et al. 2004; Almon et al. 2005a; Almon et al. 2005c). Literature searches of the 653 probe sets identified in the acute skeletal muscle dataset yielded 29 probe sets representing 22 genes whose expression is related to insulin resistance in skeletal muscles (Almon et al. 2005b). Sufficient information from our own work and from the literature allowed us to develop four rational mechanistic models describing the regulation of the expression of six genes related to insulin resistance. Data from both acute and chronic studies were simultaneously modeled.

## Dynamic Modeling

### Kinetics

The kinetics of plasma MPL following intravenous drug injection and infusion was described by a two-compartment model with a zero-order input k_0_ into the central compartment.

(1) $$\frac{{dA}_{p}}{dt}=k_{0}+k_{21}\cdot A_{t}-k_{12}\cdot A_{p}-\left(\frac{CL}{V_{p}}\right)\cdot A_{p}$$

(2) $$\frac{{dA}_{t}}{dt}=k_{12}\cdot A_{p}-k_{21}\cdot A_{t}\;\;\;\text{where }\;\;A_{p}=C_{p}\cdot V_{p}$$

where A_p_ and A_t_ are the drug amounts in the plasma and tissue compartments and *CL*, *V*_p_, *k*_12_ and *k*_21_ are clearance, central compartment volume of distribution, and distribution rate constants. These parameters were fixed according to previous literature values (Sun et al. 1999).

### Receptor dynamics

**Figure 1:**
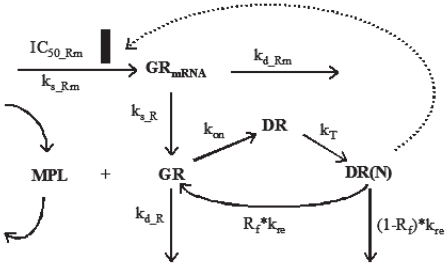
Fifth-generation CS pharmacodynamic model of receptor-gene mediated effects. Symbols and differential equations are defined in Eq. 3 to 8 and Table 1. The solid rectangle represents inhibition of glucocorticoid receptor transcription by DR(N) via an indirect mechanism.

Glucocorticoid receptor dynamics in skeletal muscle following single intravenous 50 mg/kg MPL treatment was previously described by a mechanistic receptor-gene mediated pharmacodynamic model (Fig. 1) (Sun et al. 1999). The model was developed using the assumption that unbound MPL molecules diffuse through cell membranes and bind with cytosolic free receptors. Drug-receptor complex is translocated into the nucleus where it dimerizes and binds to glucocorticoid response elements (GREs) in the target DNA. The binding of drug-receptor complex to GREs enhances or inhibits the expression of target genes. Corticosteroids are known to inhibit the expression of their own receptors (Dong et al. 1988). After dissociation from DNA, GR receptors are recycled into cytosol, where part of receptors are degraded and the rest may be further activated by MPL (Sun et al. 1999). The equations describing the model are:

(3) $$\begin{array}{l} \frac{{dGR}_{mRNA}}{dt}=k_{s\_Rm}\\ \times \left(1-\frac{DR(N)}{{IC}_{50\_Rm}+DR(N)}\right)-k_{d\_Rm}\cdot {GR}_{mRNA} \end{array}$$

(4) $$\begin{array}{l} \frac{dGR}{dt}=k_{s\_R}\cdot {GR}_{mRNA}+R_{f}\cdot k_{re}\cdot DR(N)\\ -k_{on}\cdot C_{MPL}\cdot GR-k_{d\_R}\cdot GR \end{array}$$

(5) $$\frac{dDR}{dt}=k_{on}\cdot C_{MPL}\cdot GR-k_{T}\cdot DR$$

(6) $$\frac{dDR(N)}{dt}=k_{T}\cdot DR-k_{re}\cdot DR(N)$$

where symbols represent cytosolic free glucocorticoid receptor density (*GR*), *GR mRNA* (GR*_mRNA_*), cytosolic GR-drug complex (DR), GR-drug complex in nucleus (*DR*(*N*)), zero-order *GR mRNA* synthesis rate constant (*k_s_*___*_Rm_*), and first-order rate constants for GR mRNA degradation (*k_d_Rm_*), *GR* synthesis (*k_s_R_*) and GR degradation (*k_d_*___*_R_*). Other rate constants include: second-order association rate constant of GR and drug (*k_on_*), translocation rate constant of DR from cytosol to nucleus (*k_T_*), and the overall turnover rate of DR(N) (*k_re_*). The fraction of GR that is recycled from nucleus to cytosol (*R_f_*) can be further activated by association of drug. Thus (1−*R_f_*) is the fraction of GR that is degraded during one cycle of drug-receptor complex translocation. The *IC*_50__*_Rm_* is the concentration of *DR*(*N*) at which the synthesis rate of GR mRNA is reduced to 50% of its baseline level.

Assuming the receptor system is at steady-state before administration of MPL and there is no endogenous glucocorticoid present in ADX rats, the following conditions are derived from Eq. (3) and (4).

(7) $$k_{s\_Rm}=k_{d\_Rm}\cdot {GR}_{mRNA}^{0}$$

(8) $$k_{s\_R}=k_{d\_R}\cdot \frac{{GR}^{0}}{{GR}_{mRNA}^{0}}$$

where *GR^0^_mRNA_* and *GR*^0^ are the baseline levels of GR mRNA and free cytosolic GR at time zero. In our previous study, these were measured in tissues from the same animals by quantitative Northern hybridization and [^3^H]-dexamethasone ligand binding assays (Sun et al. 1999). In the present report, *GR^0^_mRNA_* and *GR*^0^ were fitted as well as other parameters in the receptor dynamics model. Values of *DR*^0^ and *DR*(*N*)^0^ were assumed to be zero in the absence of steroid. Parameters estimated from the modeling of acute data were used to simulate receptor mRNA and receptor density profiles for the chronic study.

### Insulin receptor substrate-1 (IRS-1)

**Figure 2:**
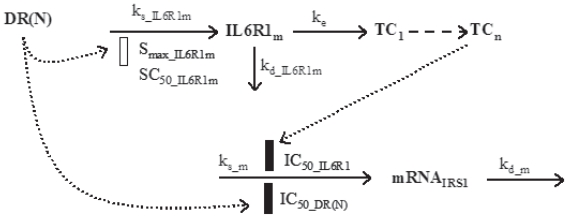
Pharmacodynamic/pharmacogenomic model for CS effects on gene expression of IL-6 receptor 1 and IRS-1. Symbols and differential equations are defined in Eq. 9 to 13 and Table 2. The solid rectangle represents inhibition while the open rectangle represents stimulation in all diagrams.

IRS-1 belongs to the IRS family of adaptor proteins and is involved in insulin signaling. It has been reported that chronic CS treatment down-regulates the expression of IRS-1 in skeletal muscle (Giorgino et al. 1993). Additionally, interleukin 6 (IL-6) exerts long-term inhibitory effects on the transcription of IRS-1 while IL-6 receptor expression is enhanced by CS treatment (Rotter et al. 2003). A model is proposed according to these regulatory pathways (Fig. 2).

The equation describing the up-regulation of IL-6 receptor type 1 mRNA (*IL*6*R*1*_m_*) time profile following MPL treatment is:

(9) $$\begin{array}{l} \frac{dIL6R1_{m}}{dt}\\ =k_{s\_IL6R1m}\cdot \left(1+\frac{S_{\text{max}\_IL6R1m}\cdot DR(N)}{{SC}_{50\_IL6R1m}+DR(N)}\right)\\ -k_{d\_IL6R1m}\cdot IL6R1_{m} \end{array}$$

where rate constants represent zero-order synthesis rate (*k*_s_IL6R1m_) and first-order degradation (*k_d_*___*_IL6R_*_1_*_m_*) of IL-6 receptor 1 mRNA. The enhancement of gene transcription is dependent on DR(N) with a maximum stimulatory effect (*S_max_*___*_IL6R_*_1_*_m_*) and *SC_50_*___*_IL6R_*_1_*_m_* is the concentration of *DR*(*N*) that leads to 50% of maximum stimulation of mRNA transcription. The baselines of IL6R1m were fixed to 1 in the modeling for both acute and chronic studies.

The IRS-1 mRNA (mRNA) was described as:

(10) $$\frac{{dTC}_{1}}{dt}=k_{e}\cdot (IL6R1_{m}-{TC}_{1})$$

(11) $$\frac{{dTC}_{n}}{dt}=k_{e}\cdot ({TC}_{n-1}-{TC}_{n})$$

(12) $$\begin{array}{l} \frac{dmRNA}{dt}=k_{s\_m}\\ \times \left(1-\frac{DR(N)}{{IC}_{50\_DR(N)}+DR(N)}\right)\\ \times \left(1-\frac{{TC}_{n}}{{IC}_{50\_IL6R1}+{TC}_{n}}\right)-k_{d\_m}\cdot mRNA \end{array}$$

where symbols represent number of transit compartments (*N*), the nth transit compartment (*TC_n_*), the first-order rate constant describing transit compartment turnover (*k_e_*), and the zero-order synthesis rate (*k_s_*___*_m_*) and first-order degradation rate (*k_d_*___*_m_*) of mRNA. The *IC*_50__*_DR_*_(_*_N_*_)_ represents the concentration of DR(N) at which the production rate of mRNA is reduced to 50% of its baseline level under the inhibition of DR(N). The *IC*_50__*_IL_*_6_*_R_*_1_ reflects the concentration of *TC_n_* at which the production rate of mRNA is one-half of its baseline level when it is only inhibited by IL-6. Assuming steady-state at time zero, Eq. 12 allows calculation of:

(13) $$k_{s\_m}=\frac{k_{d\_m}\cdot ({IC}_{50\_IL6R1}+{TC}_{n}^{0})}{{IC}_{50\_IL6R1}}$$

where *TC_n_*^0^ is the baseline value of *TC_n_* and is fixed as 1.

### Uncoupling protein 3 (UPC3) and pyruvate dehydrogenase kinase isoenzyme 4 (PDK4)

**Figure 3:**
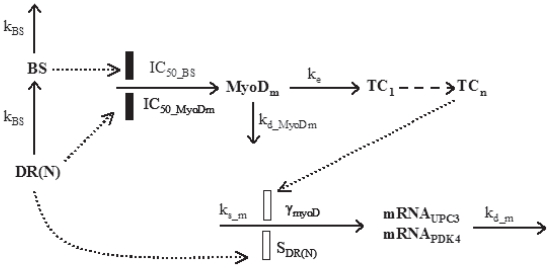
Pharmacodynamic/pharmacogenomic model for CS effects on gene expression of MyoD and its regulated genes. Symbols and differential equations are defined in Eq. 14 to 18 and Table 3.

Dexamethasone leads to increased expression of UCP3 in skeletal muscle in rats and mice (Gong et al. 1997; Sun et al. 2003). Similarly, PDK4 mRNA and protein are augmented following dexamethasone treatment and are associated with decreased glucose oxidation rates (Qi et al. 2004). It has been reported that the expression of UCP3 in muscle is enhanced by MyoD (Solanes et al. 2000; Solanes et al. 2003). Examination of the promoter regions of both UCP3 and PDK4 reveals several GREs and MyoD binding sites that are conserved in several species. Models were thus proposed based on assumptions that both UCP3 and PDK4 are regulated directly by CS and indirectly by MyoD (Fig. 3). The equation describing MyoD mRNA (*MyoD_m_*) profile is:

(14) $$\frac{dBS}{dt}=k_{BS}\cdot (DR(N)-BS)$$

(15) $$\begin{array}{l} \frac{dMyoD_{m}}{dt}=k_{s\_MyoDm}\\ \times \left(1-\frac{DR(N)}{{IC}_{50\_MyoDm}+DR(N)}\right)\\ \times \left(-\frac{BS}{{IC}_{50\_BS}+BS}\right)1-k_{d\_MyoDm}\cdot MyoD_{m} \end{array}$$

where zero-order synthesis rate (*k_s_*___*_MyoDm_*) and first-order degradation rate constants (*k_d_*___*_MyoDm_*) of MyoD mRNA occur. An intermediate biosignal (*BS*) is described by a production and degradation rate constant of k_BS_ (Jin et al. 2003). The inhibition of gene transcription is dependent on *DR*(*N*) and BS with inhibitory coefficients (*IC*_50__*_MyoDm_* and *IC*_50__*_BS_*) reflecting corresponding concentrations leading to 50% mRNA transcription reduction. The baselines of *MyoD_m_* in acute (*BmRNA_a_*___*_MyoD_*) and chronic (*BmRNA_c_*___*_MyoD_*) studies were estimated in the modeling.

(16) $$\frac{{dTC}_{1}}{dt}=k_{e}\cdot ({MyoD}_{m}/{BmRNA}_{a(c)\_MyoD}-{TC}_{1})$$

(17) $$\frac{{dTC}_{n}}{dt}=k_{e}\cdot ({TC}_{n-1}-{TC}_{n})$$

The UCP3 mRNA (mRNA) time profiles were described as:

(18) $$\begin{array}{l} \frac{dmRNA}{dt}=k_{s\_m}\cdot (1+S_{DR(N)}\cdot DR(N))\\ \times {\left({TC}_{n}\right)}^{\gamma_{MyoD}}-k_{d\_m}\cdot mRNA \end{array}$$

The PDK4 mRNA (mRNA) time profiles were described as:

(19) $$\begin{array}{l} \frac{dmRNA}{dt}=k_{s\_m}\cdot \left(1+\frac{S_{\text{max}}\cdot DR(N)}{{SC}_{50}+DR(N)}\right)\\ \times {\left({TC}_{n}\right)}^{\gamma_{MyoD}}-k_{d\_m}\cdot mRNA \end{array}$$

where *γ_MyoD_* is a power term representing the stimulatory efficiency on UCP3 or PDK4 gene transcription by *MyoD*. The transcription of PDK4 is dependent on *DR*(*N*) with a maximum stimulatory effect (*S*_max_) and a stimulatory constant (*SC*_50_). For UCP3, assuming *SC*_50_ is much higher than *DR*(*N*) concentrations, *S*_max_ and *SC*_50_ are combined as one parameter *S_DR_*_(_*_N_*_)_, which represents the linear coefficient describing the stimulatory efficiency of *DR*(*N*) on UCP3 mRNA production rate. Assuming steady-state at time zero, Eq. 18 and 19 allow calculation of:

(20) $$k_{s\_m}=\frac{k_{d\_m}}{{\left({TC}_{n}^{0}\right)}^{\gamma_{MyoD}}}$$

where *TC_n_*^0^ is the baseline value of *TC_n_* and is fixed as 1.

### Fatty acid translocase (FAT) and glycerol-3-phosphate acyltransferase (GPAT)

**Figure 4:**
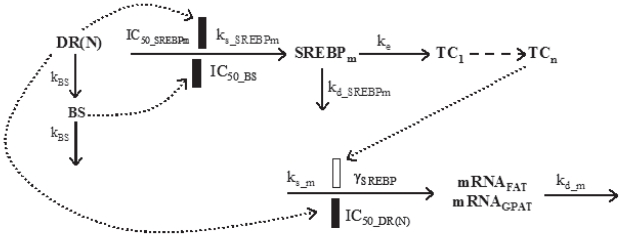
Pharmacodynamic/pharmacogenomic model for CS effects on gene expression of SREBP-1c and its regulated genes. Symbols and differential equations are defined in Eq. 19 to 24 and Table 4.

These two genes are related to carbohydrate and lipid metabolism and are involved in insulin-induced metabolic actions. They are all stimulated by sterol regulatory element binding protein-1c (SREBP-1c) (Ericsson et al. 1997; Ntambi, 1999; Nguyen et al. 2000; Almon et al. 2005b). Models are proposed to describe their time profiles according to underlying mechanisms (Fig. 4). The equations describing SREBP-1c mRNA (*SREBP_m_*) time profile following MPL treatment are:

(21) $$\frac{dBS}{dt}=k_{BS}\cdot (DR(N)-BS)$$

(22) $$\begin{array}{l} \frac{{dSREBP}_{m}}{dt}=k_{s\_SREBPm}\\ \times \left(1-\frac{DR(N)}{{IC}_{50\_SREBPm}+DR(N)}\right)\\ \times \left(1-\frac{BS}{{IC}_{50\_BS}+BS}\right)-k_{d\_SREBPm}\cdot {SREBP}_{m} \end{array}$$

where symbols represent zero-order synthesis rate (*k_s_*___*_SREBPm_*) and first-order degradation rate (*k_d_*___*_SREBPm_*) of SREBP-1c mRNA. The *IC*_50__*_SREBPm_* and *IC*_50__*_BS_* represent the concentrations of DR(N) and BS at which the corresponding production rates are reduced to 50% of baseline values. The baselines of *SREBP_m_* in acute (*BmRNA_a_*___*_SREBP_*) and chronic (*BmRNA_c_*___*_SREBP_*) studies were either fixed to 1 or estimated in the modeling.

The FAT and GPAT mRNA (mRNA) versus time profiles were described as:

(23) $$\frac{{dTC}_{1}}{dt}=k_{e}\cdot ({SREBP}_{m}/{BmRNA}_{a(c)\_SREBP}-{TC}_{1})$$

(24) $$\frac{{dTC}_{n}}{dt}=k_{e}\cdot ({TC}_{n-1}-{TC}_{n})$$

(25) $$\begin{array}{l} \frac{dmRNA}{dt}=k_{s\_m}\\ \times \left(1-\frac{DR(N)}{{IC}_{50\_DR(N)}+DR(N)}\right)\\ \times {\left({TC}_{n}\right)}^{\gamma_{SREBP}}-k_{d\_m}\cdot mRNA \end{array}$$

where *IC*_50__*_DR_*_(_*_N_*_)_ represents the concentration of *DR*(*N*) at which the production rate of mRNA is reduced to 50% of its baseline level and *γ_SREBP_* is a power term representing the stimulatory efficiency on gene transcription by SREBP-1c. FAT and GPAT mRNA data from both i.v. bolus and infusion studies were simultaneously fitted. Assuming steady-state at time zero, Eq. 25 allows calculation of:

(26) $$k_{s\_m}=\frac{k_{d\_m}}{{\left({TC}_{n}^{0}\right)}^{\gamma_{SREBP}}}$$

where *TC_n_*^0^ is the baseline value of *TC_n_* and is fixed as 1.

### Endothelin-1 (ET-1)

**Figure 5:**
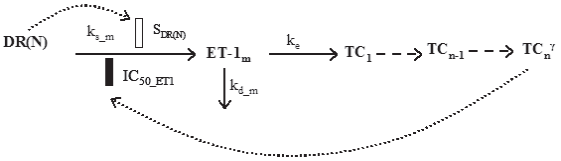
Pharmacodynamic/pharmacogenomic model for CS effects on gene expression of ET-1. Symbols and differential equations are defined in Eq. 25 to 28 and Table 5.

It has been found that ET-1 may enhance nitric oxide production via its receptor located in endothelial cells (Namiki et al. 1992). Conversely, nitric oxide can inhibit ET-1 synthesis in different cell types (Takada et al. 1996). A model is proposed assuming there is a feedback loop regulating the production of ET-1 (Fig. 5). The equations describing ET-1 mRNA (mRNA) time profile following MPL treatment are:

(27) $$\begin{array}{l} \frac{dmRNA}{dt}=k_{s\_m}\\ \times (1+S_{DR(N)}\cdot DR(N))\\ \times \left(1-\frac{{TC}_{n}}{{IC}_{50\_ET1}+{TC}_{n}}\right)-k_{d\_m}\cdot mRNA \end{array}$$

(28) $$\frac{{dTC}_{1}}{dt}=k_{e}\cdot (mRNA-{TC}_{1})$$

(29) $$\frac{{dTC}_{n}}{dt}=k_{e}\cdot ({\left({TC}_{n-1}\right)}^{\gamma }-{TC}_{n})$$

where gene transcription is dependent on *DR*(*N*) with a linear stimulatory coefficient (*S_DR_*_(_*_N_*_)_). The *IC*_50__*_ET1_* represents the concentration of TC_n_ at which the production rate of mRNA is reduced to 50% of its baseline level under the inhibition of itself and *γ* is a power term describing the amplification effect during the transit steps. Assuming steady-state at time zero, Eq. 27 allows calculation of:

(30) $$k_{s\_m}=\frac{k_{d\_m}\cdot ({IC}_{50\_ET1}+{TC}_{n}^{0})}{{IC}_{50\_ET1}}$$

where *TC_n_*^0^ is the baseline value of *TC_n_* and is fixed as 1.

### Data analysis

The gene array data were obtained from a ‘giant rat’ study design reflecting naïve pooling of data from a group of animals (Sun et al. 1999; Ramakrishnan et al. 2002). Microarray data from both i.v. bolus and infusion studies were simultaneously fitted. Since acute and infusion data were obtained from two different types of chips, a scaling factor (*SF*) was incorporated in the modeling to account for the different sensitivity of two chips to the same gene expression changes. The output functions of mRNA in acute (*Y_A_*(*t*)) and chronic (*Y_C_*(*t*)) studies were expressed as:

(31) $$Y_{A}(t)={mRNA}_{A}(t)$$

(32) $$Y_{C}(t)={\left({mRNA}_{C}(t)\right)}^{SF}$$

where *mRNA_A_*(*t*) and *mRNA_C_*(*t*) represent mRNA expression at time t in acute and chronic studies. Since relative sensitivity was considered in the present report, the scaling factor was only added to the chronic data.

Assuming that the errors from the observed and predicted responses are normally distributed, the ADAPT II program (D’Argenio and Schumitzky, 1997) with the maximum likelihood method was applied for all fittings. The following variance model was used:

(33) $$V_{i}=V\left(\theta ,\sigma ,t\right)={\left(\sigma_{1}+\sigma_{2}\cdot Y\left(\theta ,t_{i}\right)\right)}^{2}$$

where *V_i_* is the variance of the response at the *i*th time point, *t_i_* is the actual time at the *i*th time point, θ represents the systemic parameter vector for the respective pharmacodynamic (PD) model, *σ* is defined as the vector of variance parameters for the model, *Y*(θ, *t_i_*) is the predicted response value at time *t_i_* from the model. Variance parameters *σ*_1_ and *σ*_2_ were estimated along with model parameters during fittings. Mean data from 3 or 4 animals at each time point were used in fitting the models. The goodness-of-fit criteria included visual inspection of the fitted curves, estimator criterion value, sum of squared residuals, Akaike information criterion, Schwartz criterion, and coefficients of variation (CV) of the estimated parameters.

## Results

### Drug kinetics

**Figure 6:**
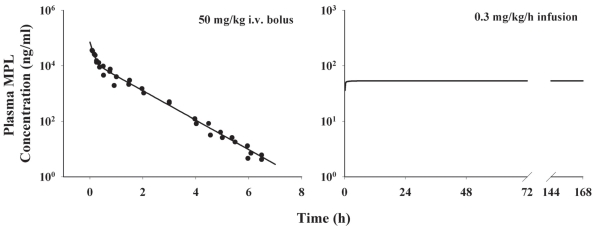
MPL pharmacokinetics following 50 mg/kg MPL i.v. bolus administration and 0.3 mg/kg/h 7-day infusion. Chronic PK profile was simulated (Eq. 1 and 2) using pharmacokinetic parameters listed in Table 1. Original plasma MPL concentrations were obtained from Sun et al. (Sun et al. 1999).

Plasma concentrations of MPL following i.v. bolus were described by a two-compartment model. The MPL PK profile following drug infusion was generated using the reported parameters (Ramakrishnan et al. 2002) (Fig. 6). The estimated and fixed parameters are summarized in Table 1.

### Receptor dynamics

**Figure 7:**
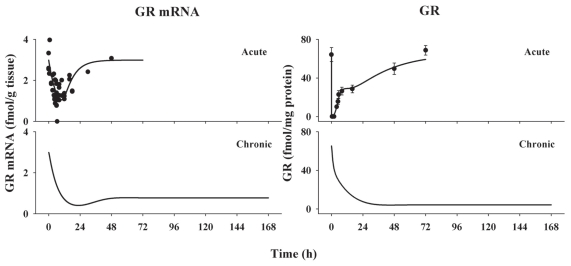
Time courses of GR mRNA and free receptor density in ADX rat muscle following 50 mg/kg MPL i.v. bolus and 0.3 mg/kg/h infusion treatments. Data points are observed values and error bars are standard deviations at each time point. Solid lines are the fitted curves for acute study or simulated curves for chronic study. Chronic profiles were simulated using parameters listed in Table 1. Original GR mRNA and cytosolic GR density data were obtained from Sun et al. (Sun et al. 1999).

The integrated receptor-gene mediated model adequately captured both *GR mRNA* and cytosolic free receptor data following acute MPL treatment. The profiles of *GR mRNA* and cytosolic *GR* density versus time and the least-squares fitted curves are shown in Figure 7. The *GR mRNA* concentration drops from the baseline level to the trough in 10 h and then slowly returns to the baseline. Free *GR* disappears quickly from cytosol and is not detectable between 0.5 to 2 h after dosing. The recovery of *GR* density shows an initial rapid phase between 2 to 20 h which is responsible for 40% of recovery and a later slower phase persisting until 72 h. Simulations of major components in the receptor dynamic model (data not shown) suggest that *DR*(*N*) increases after MPL treatment and reaches a peak at 0.1 h and declines thereafter. The *DR*(*N*) disappears 10 h after dosing. In the present report, the simulated profile of *DR*(*N*) served as the driving force for stimulation or inhibition of downstream gene transcription. Since receptor *mRNA* and receptor density data were absent for the infusion study, the resultant parameters from the acute study were used to simulate receptor dynamics in muscle during the infusion and receptor dynamics were fixed in the subsequent gene regulation modeling (Fig. 7). These simulations suggest that both receptor *mRNA* and free cytosolic receptor are maintained at very low levels during long-term MPL infusion, which is consistent with our measurements in the liver from these same animals (Ramakrishnan et al. 2002).

The fixed and estimated parameters of the receptor dynamics model are listed in Table 1. This model is over-parameterized leading to the necessity of fixing the *k_T_* value, which was derived from in vitro literature data. Translocation of GR from cytoplasm to nucleus is complete in 10 minutes when cells are incubated with 10 nM dexamethasone (Htun et al. 1996). Dexamethasone at 10^−6^ M leads to complete translocation in 0.1 h (Hache et al. 1999). However, the translocation rate in those in vitro studies is determined by both receptor-steroid binding and first-order cytosol-nucleus translocation. According to literature data, the first-order translocation rate *k_T_* has to be higher than 35 h^−1^. Our *k_T_* was fixed to 90 h^−1^ in the modeling of receptor dynamics. The *GR_mRNA_*^0^ and *GR*^0^ were estimated at 2.99 fmol/g tissue and 65.3 fmol/mg protein. The estimated *GR mRNA* and *GR* degradation rates yield half-lives of GR mRNA of 5.0 h and protein of 19.5 h. The *IC*_50__*_Rm_* was estimated at 0.911 fmol/mg protein, suggesting *GR mRNA* transcription is sensitive to the inhibition by steroid treatment. About 72% of *DR*(*N*) is recycled back into cytosol while the rest is degradated during one cytosol-nucleus cycle.

### IL6R and IRS-1

**Figure 8:**
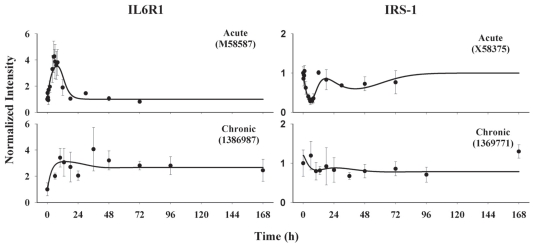
Time courses of IL-6 receptor 1 and IRS-1 gene expression in ADX rats following bolus and infusion MPL treatments. Symbols are the mean gene array data, bars are standard deviations, and solid lines are fitted curves (Eq. 10–12).

Simultaneous modeling using a basic indirect response model with direct stimulation by *DR*(*N*) well captured IL-6 receptor 1 mRNA following both i.v. bolus and infusion treatments. Figure 8 shows the normalized data from gene arrays and the fitted curves. Following 50 mg/kg MPL treatment, expression of IL-6R1 *mRNA* exhibits a similar pattern as tyrosine aminotransferase (TAT) in the liver (Sun et al. 1998; Jin et al. 2003). It shows a rapid induction of expression which peaked at 5.5 h following drug treatment. The enhanced expression lasts for about 20 h and thereafter maintains baseline level. Compared to *DR*(*N*), the induction of IL-6 receptor mRNA reveals a 3 h time delay. Following the 0.3 mg/kg/h infusion, IL-6 expression is increased and maintained at a high level throughout the long-term treatment period. Table 2 lists the estimated PD parameters and their precision. The estimated *k_d_*___*_IL_*_6_*_R_*_1_*_m_* yields a degradation half-life of 2.26 h. The stimulatory constant *SC*_50__*_IL_*_6_*_R_*_1_*_m_* was estimated at 1.09 fmol/mg protein, suggesting high sensitivity to MPL action.

The transcription of IRS-1 was described by a more complex regulation with inhibitory effects derived directly by *DR*(*N*) and indirectly through IL-6 receptor. The proposed PK/PD model reasonably captured the down-regulation of IRS-1 message by MPL administration (Fig. 8). In the acute study, the expression profile exhibits two inhibitory phases. The first decline occurs immediately after drug treatment and reaches the nadir around 5.5 to 6 h. This phase was successfully described by direct inhibition by *DR*(*N*) under the assumption that the drug-receptor complex represses the transcription of IRS-1 mRNA. It returns to a value which is close to baseline by 12 h. The second decline phase starts at 18 h and exhibits a much slower decrease with a trough at 40 h. The second phase was described by augmented inhibition of IRS-1 mRNA transcription by the IL-6 receptor. Comparing the curves of IRS-1 and IL-6 receptor 1 mRNA reveals a delay time of 30 h between the second decline of IRS-1 and its driving force. This is consistent with a previous report that reduction of IRS-1 mRNA is seen 24 h following IL-6 treatment in adipose cells (Rotter et al. 2003). The long time delay necessitated the utilization of multiple transit compartments between the driving force and the regulated gene expression. Five transit compartments were adequate to capture the curve and give reasonable parameter estimates and CV values (Table 2). In the infusion study, IRS-1 mRNA declines following drug treatment and reaches a steady-state throughout the 7-day study.

### MyoD, UCP3 and PDK4

**Figure 9:**
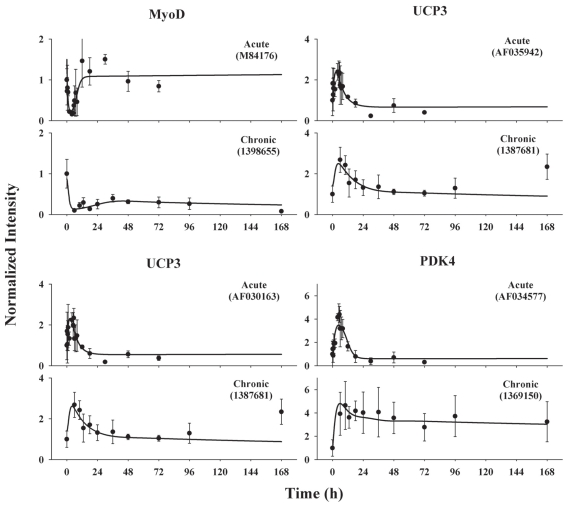
Time courses of MyoD and its regulated genes UCP3 (represented by two probe sets) and PDK4 in ADX rats following bolus and infusion MPL treatments. Symbols and lines are defined as in Figure 8.

MyoD mRNA expression profiles following i.v. bolus and infusion treatments were well captured by the dynamic model assuming a direct inhibition of transcription by *DR*(*N*) and another inhibitory effect from the biosignal (Fig. 9). In the acute study, MyoD mRNA exhibits a sharp decline immediately following MPL treatment. The trough occurs at 4 h, only 2 h after the peak of *DR*(*N*). Following infusion, MyoD transcription quickly declined and reached the first trough at 6 h and was reduced to almost zero. Then it increased with a slow slope and regained 40% of its basal level at 36 h and then decreased thereafter. The second decline is described by the inhibitory effect from the biosignal. The estimated parameters are listed in Table 3. A high *k_d_*___*_MyoDm_* yields a short degradation half-life of 0.495 h^−1^ and is consistent with the rapid decline and short time delay. The parameters were estimated with reasonable precision with most CVs below 30% (Table 3).

The proposed model was able to describe the time profiles of both PDK4 and two probe sets for UCP3. The original data and the fitted curves are shown in Figure 9. In the acute study, both UCP3 and PDK4 mRNA reveal a sharp enhancement of production which is well captured by the direct stimulation of *DR*(*N*). The peaks occur at 5 h after drug administration. All three probe sets return to baseline by 15 h and thereafter continuously decline and reach steady-states after 18 h. The inhibitory phases were adequately captured by the reduced activation by MyoD. Following drug infusion, all three probes exhibit only enhanced transcription without the inhibitory phase. One transit compartment was adequate for modeling of both UCP3 and PDK4. The degradation half-life of UCP3 was estimated between 3.6 and 4.4 h while that of PDK4 was 2.1 h. Moderate stimulatory coefficients (*S_DR_*_(_*_N_*_)_) around 0.15 (fmol/mg protein)^−1^ were obtained for UCP3. For PDK4, two parameters *S*_max_ and *SC*_50_ were required to capture both acute and chronic profiles simultaneously. The estimated low *SC*_50_ suggests high efficiency of the stimulatory effect and also justifies the requirement of using both parameters. Two probe sets for UCP3 exhibit similar expression versus time patterns and yield similar parameter estimates, suggesting good reproducibility of the oligonucleotide microarray assay. Most parameters were estimated with CV below 40% (Table 3).

### SREBP-1c, FAT, and GPAT

**Figure 10:**
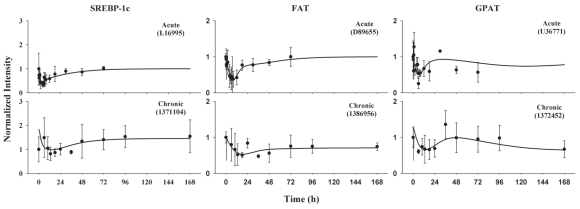
Time courses of SREBP-1c and its regulated genes FAT and GPAT in ADX rats following bolus and infusion MPL treatments. Symbols and lines are defined as in Figure 8.

The SREBP-1c mRNA time profiles were captured by the joint inhibitory effects on the transcription directly by *DR*(*N*) and indirectly by an unknown intermediate biosignal (Fig. 10). The expression of SREBP-1c is down-regulated after MPL treatment. Following its trough at 4 h in the acute study, it returned to baseline in a biphasic pattern with an initial fast return phase and a later shallow phase. A similar pattern was shown in the chronic study. The biphasic change suggests that two driving forces probably are involved. The mediator BS was incorporated in the model based on the assumption that the absolute change of a regulator level by *DR*(*N*) may elicit an inhibitory effect on the transcription rate of SREBP-1c mRNA (Jin et al. 2003). The estimated *k_BS_* of 0.0436 h^−1^ yields a relatively long mean transit time of 23 h, which is consistent with the prolonged repression of SREBP-1c until 72 h. The degradation half-life of SREBP-1c mRNA was fixed to 1 to obviate over-parameterization. Both *DR*(*N*) and the biosignal display moderate inhibition on SREBP-1c transcription with an *IC*_50__*_SREBP_* of 66.7 fmol/mg protein and an *IC*_50__*_BS_* of 12.4 fmol/mg protein. The estimated parameters are listed in Table 4. The dynamics of SREBP-1c served as the driving force for FAT and GPAT and was fixed in the following modeling.

Simultaneous modeling using the proposed model well captured the expression of FAT and GPAT (Fig. 10). They were down-regulated following 50 mg/kg MPL with the first troughs between 5.5 to 8 h. The initial declines were well described by the direct inhibition of gene transcription by *DR*(*N*) for both genes. The suppressed activation of SREBP-1c on GPAT leads to a second decline while the same regulatory mechanism leads to a prolonged return phase for FAT. Following 7-day MPL infusion, the first trough occurs around 20 h for both genes. Similarly to what was seen in the acute study, a second decline occurs only with GPAT but not with FAT. The degradation rate constants of the messages are around 0.16 h^−1^. The *DR*(*N*) has a moderate to high inhibitory effect on the gene transcription with the *IC*_50__*_DR_*_(_*_N_*_)_ between 2.27 to 9.97 fmol/mg protein. GPAT has a higher *γ_SREBP_* than that of FAT which may explain the stronger suppression of GPAT message under the control of SREBP-1c. Two to four transit compartments were required to fit the data. The estimated parameters and their precisions are shown in Table 4.

### ET-1

**Figure 11:**
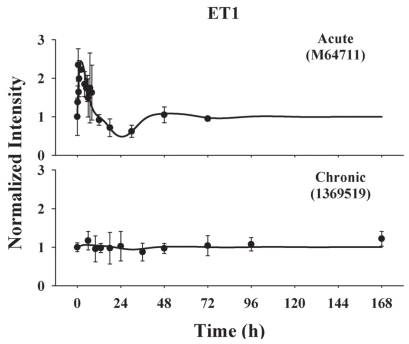
Time course of ET-1 gene expression in ADX rats following. Bolus and infusion MPL treatments. Symbols and lines are defined as in Figure 8.

The time profiles of ET-1 mRNA following both acute and chronic treatments were captured by the proposed model (Fig. 11). Immediately following 50 mg/kg MPL dosing, it exhibits a sharp induction peak at 2–3 h and then decreases below baseline after 12 h. The abrupt stimulation and the short time shift between ET-1 mRNA and *DR*(*N*) can be explained by a fast mRNA degradation rate constant of 0.958 h^−1^, yielding a half-life of 0.723 h. A short half-life might be necessary to obtain quickly induced or reduced effects on vasoconstriction. The power parameter *γ* was estimated at 8.23, representing a considerable magnifying effect during the signaling transduction steps. A model with 8 transit compartments was selected based on visual inspection of the fitted curve and other model selection criteria. Following infusion, the change of ET-1 mRNA expression was flat. This apparent lack of response of ET-1 to chronic infusion might be due to low sensitivity of the probe set on genechip 230A to the change of mRNA level, which can be seen from the estimated SF of 0.118. Table 5 summarizes the estimated parameters and their precision.

## Discussion

Insulin resistance is a pathological state where peripheral tissues, particularly skeletal muscle, fail to respond to circulating insulin (Gual et al. 2005). One of the earliest abnormalities observed in skeletal muscle is the reduced insulin-induced uptake of glucose. The 50 mg/kg MPL i.v. injection in ADX rats leads to a transient increase in the plasma glucose concentration that begins about 2 h and lasts for 9 h (Almon et al. 2005b). Plasma insulin concentrations are increased between 2 to 48 h after dosing, probably due to increased plasma glucose levels.

The development and progression of insulin resistance is not mediated by a single gene. Abnormalities in multiple molecules and tissues are involved in the pathogenesis of the disease. In such situations, research focused on single mediators may distort the true underlying molecular and cellular mechanisms and inadequately reveal overall regulatory factors and pathways. By analyzing joint temporal responses of many genes simultaneously, it should be possible to gain a better understanding of regulatory pathways and their contributions to this complex pathology. By using two different dosing regimens in the time series format, we not only obtained joint confirmation but also augmented the elucidation of underlying mechanisms of regulation. In the present report we describe the development of four models that encompass six genes associated with insulin resistance. These models include both direct effects of CS on the genes as well as indirect effects due to the effects of CS on other transcription factors such as MyoD and SREBP-1c.

The first model describes the regulation of IRS-1 which plays a central role in insulin signaling. IRS-1 is phosphorylated in response to insulin, insulin like growth factor-1, and cytokines and is preferentially involved in the metabolic actions of insulin (Gual et al. 2005). A decline of IRS-1 amount or function has been linked to decreased glucose uptake in insulin resistant animal models and type II diabetic patients (Sesti et al. 2001; Smith, 2002).

Strong correlations between local and circulating proinflammatory cytokines and insulin resistance have been reported (Senn et al. 2002). Among them, IL-6 has the strongest correlation with insulin resistance and type II diabetes. In liver, IL-6 elevates hepatic glucose output and increases blood glucose by interfering with insulin-induced kinase cascades such as tyrosine phosphorylation of IRS-1 (Senn et al. 2002). Adipose tissue and muscle, two peripheral tissues that are responsible for glucose disposal, are important sites of IL-6 production (Senn et al. 2002). Thus it is possible that IL-6 produced in these tissues may act at the local site and interfere with insulin-induced signaling. It has been found that IL-6 exerts long-term inhibitory effects on the transcription of IRS-1 (Rotter et al. 2003). Although it is well accepted that CS also inhibit the expression of many cytokines including IL-6 in inflammation, it is not known if CS exhibits similar effects in healthy animals. In our studies, the expression of IL-6 mRNA in muscle did not show significant change. However, we observed a significant up-regulation of IL-6 receptor type 1 transcription. Thus it is reasonable to assume that increased expression of IL-6 receptor potentially enhances muscle sensitivity to IL-6 and augments the inhibitory effect of IL-6 on IRS-1 transcription.

The second model describes the regulation of two nuclear encoded mitochondrial genes that have been associated with insulin resistance, UCP3 and PDK4. Uncoupling proteins are a family of transporters that localize in the mitochondrial inner membrane and dissipate the transmembrane potential by transporting protons from the inter-membrane space back into the matrix (Garvey, 2003). UCP3 exhibits limited tissue-specific expression confined to skeletal muscle in humans. Accumulating evidence indicates that in skeletal muscle, UCP3 contributes to lipid uptake by mitochondria rather than uncoupling oxidative phosphorylation (Garvey, 2003). PDK4 is an enzyme that inactivates the mitochondrial pyruvate dehydrogenase complex. It thus reduces glucose utilization by skeletal muscle, preventing pyruvate (the product of glycolysis) from being used by the mitochondria (Sugden, 2003). The PDK4 gene is preferentially expressed in skeletal muscle. Both genes are associated with enhanced fatty acid oxidation, thus probably are involved in the preferential fuel utilization of lipids instead of glucose in insulin resistance states. Enhanced expressions of both UCP3 and PDK4 in skeletal muscle have been associated with obesity and type II diabetes in humans (Bao et al. 1998; Sugden and Holness, 2002).

The bi-directional profiles of UCP3 and PDK4 mRNA expression following MPL treatment suggest that more than one mediator is involved in the regulatory pathways. The down-regulation of MyoD by CS that we observed in our studies along with literature searches and examination of the promoter regions of the two genes indicate that myogenic factor MyoD might be involved in a complex signaling network (Solanes et al. 2000; Solanes et al. 2003). MyoD belongs to the basic helix-loop-helix family of DNA-binding transcription factors. It is a master regulator of muscle lineage differentiation and is responsible for the preferential expression of skeletal muscle-specific genes (Solanes et al. 2000). At least two phylogenetically conserved MyoD responsive sites were found in the promoter regions of both genes. MyoD is required not only for UCP3 promoter activity but also for its sensitivity to activation by other ligands (Solanes et al. 2000; Solanes et al. 2003). Additionally, the muscle-preferential expression of UCP3 and PDK4 also supports an essential role of MyoD in transcriptional regulation.

The third model describes the regulation of the expression of two genes involved in lipid metabolism in skeletal muscle. The first is FAT, also called CD36, which is a membrane protein that facilitates fatty acid uptake and use by skeletal muscle (Heron-Milhavet et al. 2004). Muscle-specific over expression of FAT reverses insulin resistance and diabetes in animal models (Heron-Milhavet et al. 2004). The second is GPAT which catalyzes the initial and committed step in the biosynthesis of triglycerides and phospholipids (Ericsson et al. 1997). Expression of these genes indicates a shift of energy consumption in the skeletal muscle from glycolysis towards β-oxidation.

SREBP-1c is a transcription factor of the basic helix-loop-helix/leucine zipper family. It controls expression of genes which are related to adipogenesis and fatty acid metabolism as well as cholesterol metabolism (Nguyen et al. 2000). SREBP-1c is transcriptionally induced by insulin and its enhanced expression mediates the transcriptional effects of insulin in skeletal muscle (Guillet-Deniau et al. 2002). It has been observed that over-expression of SREBP-1c mimics the effects of insulin such as stimulation of glycolytic and lipogenic enzymes. It exerts a pivotal role in long-term muscle insulin sensitivity. The prolonged returning of FAT expression and the second trough of GPAT at 48 h raises the possibility that at least one intermediate biosignal is involved. Both genes are transcriptionally induced by SREBP-1c (Ericsson et al. 1997; Ntambi, 1999; Nguyen et al. 2000; Almon et al. 2005b). The addition of SREBP-1c as an intermediate driving force was able to capture the time profiles of FAT and GPAT following both acute and infusion dosing regimens.

ET-1 is a potent vasoconstrictor. It has been observed that many organs such as heart, lung and skeletal muscles synthesize ET-1, which may act locally to regulate blood flow (Sakurai et al. 1991; Guo et al. 1998). The ET-1 detected in this study might be muscle in origin, endothelial cell in origin, or both. Elevated muscle ET-1 may reduce blood flow to the musculature and thus reduce glucose disposal in skeletal muscle (Santure et al. 2002). Reciprocal regulation of ET-1 and nitric oxide, a vasodilator have been demonstrated and summarized by Rossi et al. (Rossi et al. 2001). The production of nitric oxide is activated by ET-1 while the elevated nitric oxide level is able to inhibit ET-1 synthesis (Namiki et al. 1992; Takada et al. 1996). This forms a negative feedback loop which may offer an oscillatory feature to ET-1 expression pattern once it is induced or inhibited by drug treatment. The fitted curve in our acute study exhibited a fluctuation around the baseline. Other studies demonstrate that ET-1 also exhibits similar time profiles following interleukin-1β and lipopolysaccharide treatments in human endothelial cells with an initial up-regulation and a later down-regulation at 24 h (Zhao et al. 2001; Zhao et al. 2003). Those results suggest that this oscillatory feature of ET-1 is not specific to CS treatment. ET-1 expression following drug infusion did not show significant change, possibly due to reduced probe set sensitivity in the chip used for the chronic study.

In the present report, most modeled time profiles feature long time delays between the intermediate biosignal and the regulated changes. One or more transit compartments were incorporated in the models to account for the delay. Following drug treatment, the resultant changes in message have to translate into protein changes before downstream regulatory steps can continue. Other steps such as translocation in or out of the nucleus or induction of other mediators might also be involved.

Time series studies of gene expression following CS treatment shows that many genes such as UCP3, PDK4, and ET-1 exhibit both induction and suppression depending on time. Evaluation of drug effects at a single time point may not reveal the overall effects of the drug. In such situations, time series design and mathematical modeling provide a useful approach to explore the diverse effects at different times and the actual or potential underlying mechanisms.

It is interesting to see that genes with common regulators such as UCP3 and PDK4 display comparable expression versus time patterns. This may suggest shared or similar signaling pathways associated with the regulation of these genes. However, genes regulated by common mediators may also exhibit distinct patterns, possibly due to diverse signaling pathways or differences in parameter values.

The sampling time points in the acute studies were selected based on prior knowledge of the CS-induced TAT expression profile following the same acute dosing regimen. The expression of TAT reaches a peak at 6 h following acute dosing and returns to baseline at 18 h (Sun et al. 1998). Such a sampling strategy well characterized the earlier expression changes of most genes which are rapidly induced or repressed by steroids. However, the sparse sampling after 18 h leads to lack of information to characterize slower, more complex signaling events, especially for those genes that are controlled by multiple regulatory cascades. Two genes involved in fatty acid metabolism, glycerol-3-phosphate dehydrogenase (GPDH) and stearyl-CoA desaturase 2 (SCD2) also exhibited a second decline at 48 h following MPL (Almon et al. 2005b). Both GPDH and SCD2 are activated by the master transcription factor SREBP-1c (Nguyen et al. 2000). However, lack of information after 30 h excluded the possibility of using complex models to capture their profiles. The initial sampling time point in the chronic study (6 hours) was selected to allow the drug to reach equilibrium in circulation. This may have impaired our ability to capture initial dynamics. However, the responses to two dosing regimens together clearly augment each other.

A scaling factor was used in modeling of all genes when both acute and chronic data were simultaneously fitted. Two different chips U34A and 230A were utilized for acute and chronic studies. The probe sets representing the same gene on these two chips differ in their nucleotide length and sequences. This may lead to different hybridization efficiencies and therefore different relative sensitivities to gene expression changes. In the modeling, a power scaling factor was incorporated to account for these differences.

This study was limited by the availability of genes on the chips. The assay sensitivity and our data analysis methods also restricted the number of genes that were identified by this approach, especially for low abundance message or suppressed genes. Some important regulators and biomarkers may not be included. In addition to its transcriptional regulatory effects, CS also exerts effects by affecting protein synthesis or interaction with other proteins, which cannot be detected in the current study.

In the present study we used results from two gene array time series to develop dynamic models for the regulation of several genes associated with insulin resistance in skeletal muscle. In order to begin to understand complex polygenic phenomena such as insulin resistance, it necessary to develop quantitative, experimentally testable hypotheses such as the dynamic models presented in this report.

## Figures and Tables

**Table 1:** Fixed and estimated pharmacokinetic and receptor dynamic parameters.

Parameter (unit)	Definition	Value	CV%
**Pharmacokinetics**			
CL (l/h/kg)	Clearance	2.86^a^/5.61^b^	9^a^–^b^
V_p_ (l/kg)	Central volume of distribution	0.70^a^/0.82^b^	39^a^–^b^
k_12_ (1/h)	Distribution rate constant	4.33^a^/0.32^b^	47^a^–^b^
k_21_ (1/h)	Distribution rate constant	3.09^a^/0.68^b^	15^a^–^b^
**Receptor dynamics**			
k_s_Rm_ (fmol/g muscle/h)	GR mRNA synthesis rate constant	0.416	–c
IC_50_Rm_ (fmol/mg protein)	Inhibition constant	0.911	126
k_d_Rm_ (1/h)	GR mRNA degradation rate constant	0.139	18
k_on_ (1/nmol/h)	Association rate constant	0.00269	73
k_re_ (1/h)	DR(N) overall loss rate constant	0.618	50
R_f_	Recycling fraction	0.720	12
k_d_R_ (1/h)	GR degradation rate constant	0.0356	27
k_T_ (1/h)	Translocation rate constant	90	–
k_s_R_ (fmol/mg/(fmol mRNA/g)/h)	GR synthesis rate constant	0.777	–c
GR_mRNA_^0^ (fmol/g muscle)	Baseline GR mRNA	2.99	8
GR^0^ (fmol/mg protein)	Baseline GR	65.3	3

a Estimated from i.v. bolus data.

b Fixed for infusion data.

c Secondary parameters.

**Table 2:** Estimated PD/PG parameters for IL-6 receptor 1 and IRS-1.

Parameter (unit)	Definition	Estimate	CV%
**IL-6 receptor 1**			
k_d–IL6R1m_(h^−1^)	mRNA degradation rate constant	0.306	38
S_max–IL6R1m_	Maximum stimulation by DR(N)	3.05	23
SC_50–IL6R1m_(fmol/mg protein)	Stimulatory constant by DR(N)	1.09	107
SF	Scaling factor	0.857	10
**IRS-1**			
k_e_ (h^−1^)	Transduction rate constant	0.144	18
k_d__m (h^−1^)	mRNA degradation rate constant	0.313	13
IC_50_DR_(_N_) (fmol/mg protein)	Inhibition constant by DR(N)	5.95	24
IC_50_IL6R1_	Inhibition constant by IL-6 receptor 1	0.196	182
SF	Scaling factor	0.305	18
N	Number of transit compartments	5	–

**Table 3:** Estimated PD/PG parameters for *MyoD* and its regulated genes.

Parameter (Unit)	Definition	Estimate	CV%
k_d–MyoDm_ (h^−1^)	mRNA degradation rate constant	1.40	23
IC_50–MyoDm_ (fmol/mg protein)	Inhibition constant by DR(N)	8.95	23
kBS (h−1)	Production rate constant for Biosignal	0.001	–
IC_50–BS_ (fmol/mg protein)	Inhibition constant by biosignal	0.65	46
B_mRNAa_MyoD_	MyoD mRNA baseline in acute study	1.499	14
B_mRNAc_MyoD_	MyoD mRNA baseline in chronic study	0.907	18
SF_MyoD_	Scaling factor	1.41	24

		UCP3 (AF035942)		UCP3 (AF030163)		PDK4	
		Estimate	CV%	Estimate	CV%	Estimate	CV%
k_e_ (h^−1^)	Transduction rate constant	0.819	61	0.930	63	0.242	96
k_d_m_ (h^−1^)	mRNA degradation rate constant	0.158	30	0.192	31	0.337	45
S_DR(N)_ (fmol/mg protein)^−1^	Stimulatory coefficient by DR(N)	0.148	40	0.170	40	–	–
S_max_	Maximum stimulatory effect by DR(N)	–	–	–	–	6.61	45
SC_50_ (fmol/mg protein)	Stimulation constant by DR(N)	–	–	–	–	1.8	95
*γ*_MyoD_	Power stimulatory constant by MyoD	0.829	28	0.900	28	0.48	67
BmRNA^a^	mRNA baseline in acute study	0.861	9	0.725	10	0.706	11
BmRNA^c^	mRNA baseline in chronic study	1.40	–	1.40	–	1.00	–
SF	Scaling factor	1.08	21	1.04	24	0.962	14
N	Number of transit compartments	1	–	1	–	1	–

**Table 4:** Estimated PD/PG parameters for SREBP-1c and its regulated genes.

Parameter (unit)	Definition	Estimate	CV%
k_d–SREBPm_ (h^−1^)	mRNA degradation rate constant	1.0	–
IC_50–SREBPm_(fmol/mg protein)	Inhibition constant by DR(N)	66.7	35
IC_50–BS_(fmol/mg protein)	Inhibition constant by biosignal	12.4	26
k_BS_ (h^−1^)	Biosignal production rate	0.0436	37
B_mRNAa_SREBP_	SREBP mRNA baseline in acute study	1	–
B_mRNAc_SREBP_	SREBP mRNA baseline in chronic study	1.83	8
SF_SREBP_	Scaling factor	1.01	23

		FAT		GPAT	
		
		Estimate	CV%	Estimate	CV%
k_e_ (h^−1^)	Transduction rate constant	0.122	52	0.0333	59
k_d–_m (h^−1^)	mRNA degradation rate constant	0.167	7	0.162	27
IC_50_DR(N)_ (fmol/mg protein)	Inhibition constant by DR(N)	2.27	34	9.97	64
*γ*_SREBP_	Power stimulatory constant by SREBP-1c	0.613	36	2.16	84
BmRNAa	mRNA baseline in acute study	1	–	0.951	8
BmRNAc	mRNA baseline in chronic study	1	–	1.32	17
SF	Scaling factor	0.379	13	0.864	48
N	Number of transit compartments	2	–	4	–

**Table 5:** Estimated PD/PG parameters for ET-1.

Parameter (unit)	Definition	Estimate	CV%
k_d_m_ (h^−1^)	mRNA degradation rate constant	0.958	17
S_DR(N)_ (fmol/mg protein)^−1^	Stimulatory coefficient by DR(N)	0.0313	9
k_e_ (h^−1^)	Transduction rate constant	0.343	7
I_C50_ET1_	Inhibition constant by ET-1	11.1	131
*γ*	Amplification power constant	8.23	50
SF	Scaling factor	0.118	116
N	Number of transit compartments	8	–
